# Machine Learning Approach to Predict the Probability of Recurrence of Renal Cell Carcinoma After Surgery: Prediction Model Development Study

**DOI:** 10.2196/25635

**Published:** 2021-03-01

**Authors:** HyungMin Kim, Sun Jung Lee, So Jin Park, In Young Choi, Sung-Hoo Hong

**Affiliations:** 1 Department of Medical Informatics College of Medicine The Catholic University Seoul Republic of Korea; 2 Department of Biomedicine & Health Sciences College of Medicine The Catholic University Seoul Republic of Korea; 3 Department of Urology Seoul St. Mary’s Hospital College of Medicine, The Catholic University Seoul Republic of Korea

**Keywords:** renal cell carcinoma, recurrence, machine learning, naïve Bayes, algorithm, cancer, surgery, web-based, database, prediction, probability, carcinoma, kidney, model, development

## Abstract

**Background:**

Renal cell carcinoma (RCC) has a high recurrence rate of 20% to 30% after nephrectomy for clinically localized disease, and more than 40% of patients eventually die of the disease, making regular monitoring and constant management of utmost importance.

**Objective:**

The objective of this study was to develop an algorithm that predicts the probability of recurrence of RCC within 5 and 10 years of surgery.

**Methods:**

Data from 6849 Korean patients with RCC were collected from eight tertiary care hospitals listed in the KOrean Renal Cell Carcinoma (KORCC) web-based database. To predict RCC recurrence, analytical data from 2814 patients were extracted from the database. Eight machine learning algorithms were used to predict the probability of RCC recurrence, and the results were compared.

**Results:**

Within 5 years of surgery, the highest area under the receiver operating characteristic curve (AUROC) was obtained from the naïve Bayes (NB) model, with a value of 0.836. Within 10 years of surgery, the highest AUROC was obtained from the NB model, with a value of 0.784.

**Conclusions:**

An algorithm was developed that predicts the probability of RCC recurrence within 5 and 10 years using the KORCC database, a large-scale RCC cohort in Korea. It is expected that the developed algorithm will help clinicians manage prognosis and establish customized treatment strategies for patients with RCC after surgery.

## Introduction

Renal cell carcinoma (RCC) accounts for 90% of malignant tumors in the kidney and is twice as common in men as in women [1]. Kidney cancer, therefore, generally refers to RCC. It is the sixth most frequently diagnosed cancer in men and the 10th most frequently diagnosed cancer in women worldwide [2]. According to the cancer statistics from the National Cancer Center, the number of new kidney cancer cases in Korea in 2017 was 5299, accounting for approximately 2.3% of the total of 232,255 cancer cases. Further, the incidence of kidney cancer per 100,000 people has been increasing since 1999 [3]. RCC is one of the most lethal types of malignant tumors in urology, with approximately 20% to 30% of patients with RCC suffering from metastatic diseases, and more than 40% of patients eventually die of the disease [4-6]. The main treatment for RCC is radical nephrectomy; for small tumors, partial nephrectomy is performed to preserve kidney function [7].

RCC can be completely cured through full surgical resection if there is no evidence of preoperative metastatic disease. However, it has a high recurrence rate of 20% to 30% [8,9], and approximately 50% of recurrences occur within 2 years [8,10]. RCC recurrence is generally classified as early recurrence or late recurrence based on the 5-year threshold [11]. Most recurrences occur during the early recurrence period (within 5 years) [11,12], whereas approximately 10% occur during the late recurrence period (after 5 years) [11,13].

RCC is generally resistant to radiation and chemotherapy, making treatment of its recurrence difficult [4]. Therefore, it is necessary to predict the probability of RCC recurrence so that risk factors can be managed in advance. The Memorial Sloan Kettering Cancer Center (MSKCC) in the United States developed a nomogram that predicts the probability of recurrence within 5 years using the symptoms and histology of 601 patients with kidney cancer who received surgical treatment in 2001 [14]. Additionally, in 2005, a nomogram was developed to predict the recurrence probability within 5 years using the pathological stage, Fuhrman nuclear grade, tumor size, necrosis, vascular invasion, and clinical presentation variables of 701 patients with kidney cancer [15]. Previous studies have used small-scale RCC cohorts from single institutions, and the data have included censored data, where the values of the observations were only partially known. If censored data are included, they can be applied in the Cox proportional hazards model, a standard statistical technique for modeling censored data, but they are difficult to apply to other machine learning (ML) techniques [16].

In this study, we used a multicenter, large-scale RCC cohort collected from eight tertiary care hospitals in Korea; we removed censored data and used only the fully observed data. ML focuses on building new predictive models by performing extensive searches on multiple models and parameters and then performing validation [17]. The objective of this study was to develop an algorithm that could predict the recurrence probability of RCC after surgery within 5 and 10 years by applying eight representative ML algorithms to a large-scale Korean RCC cohort. Using the developed algorithm, clinicians can manage postoperative patient outcomes and establish personalized treatment strategies.

## Methods

### Study Population

The data used in this study were obtained from a large-scale cohort of Korean patients with RCC assembled from the KOrean Renal Cell Carcinoma (KORCC) web-based database. It consisted of 206 variables, including demographic information such as age, height, and weight, as well as pathological information, including clinical stage, pathological stage, Fuhrman nuclear grade, and survival period [18]. The study protocol was approved by the institutional review board of the Catholic University of Korea (IRB No. KC20ZIDI0966). The data of 6849 patients who participated in the KORCC study group as of July 1, 2015, were collected from eight tertiary hospitals.

### Variable Selection and Data Cleansing

The *t* test for continuous variables and the chi-square test for categorical variables were used to explore variables that significantly affect recurrence. In both tests, variables with missing values were removed to ensure that the data used were complete and without missing values. At a significance level of *P*=.05, we first extracted 31 variables showing significant differences between the recurring and nonrecurring groups. Of the 31 variables extracted, 10 variables that had significant effects on recurrence in actual clinical trials were finally extracted based on the expert advice of a urologist. The final 10 selected variables were gender, age, BMI, smoking, pathological tumor stage, histological type, necrosis, lymphovascular invasion, capsular invasion, and Fuhrman nuclear grade.

Several studies reported that age ≥60 years, Fuhrman nuclear grade ≥3, and pathological stage ≥pT2 were statistically associated with RCC recurrence [19]. In addition, women had better prognoses after surgery than men [20], and individuals with higher BMIs showed better prognoses than those with normal or lower BMIs [21]. Furthermore, the prognoses of smokers were worse than those of nonsmokers [22], and pathological variables such as histological type [23], necrosis [24], lymphovascular invasion [11], and capsular invasion [25] were all related to the recurrence of RCC.

**Figure 1:**
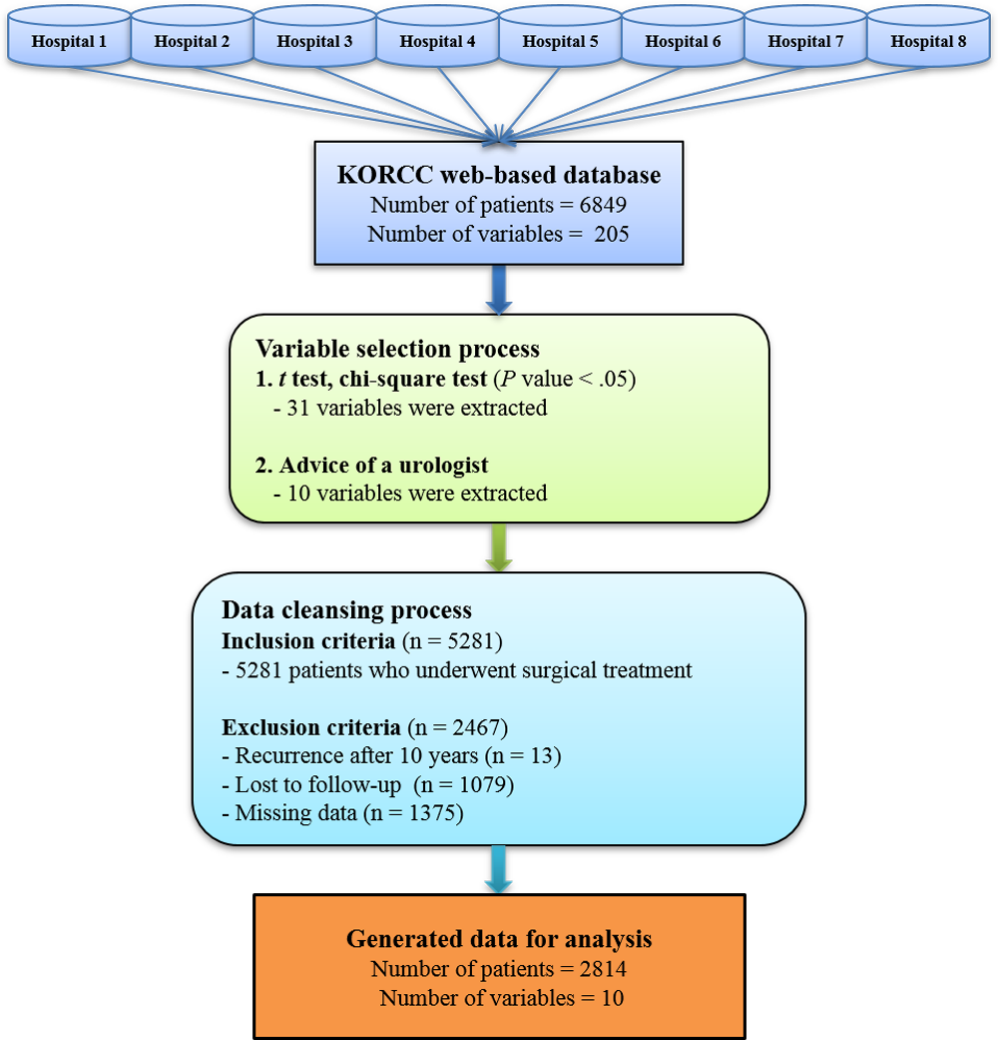
Data generation process for analysis. KORCC: Korean Renal Cell Carcinoma.

Next, we cleansed the data to present them in a form suitable for analysis. Of the 6849 patients, only 5281 patients who received surgical treatment were included in the analysis. Of those 5281 patients, 13 patients with recurrence after 10 years, 1079 lost to follow-up, and 1375 with missing values in 10 variables were excluded from the analysis. Finally, a subset of 2814 patients with values for 10 variables was available for analysis (Figure 1).

### Dealing with the Imbalanced Data Set

One of the most frequent problems in applying ML classification algorithms is data imbalance [26,27]. In the medical field, data asymmetry occurs between normal and abnormal classes because most patients are concentrated in the “normal” class, whereas relatively few—such as patients with cancer—are in the “abnormal” class. In this case, the ML algorithm attempts to improve the performance by predicting normal classes, in which most patients are concentrated, resulting in lower predictability of abnormal classes with small numbers of patients [27]. However, from a research perspective, it is more important to predict abnormal classes; hence, it is necessary to deal with the imbalanced data.

In this study, the synthetic minority oversampling technique (SMOTE) was applied to the training data set to solve the imbalance problem. SMOTE is an oversampling method that is widely used when ML is applied to data with high imbalance [28,29]. Before applying SMOTE, the ratio of patients in the recurrence group to patients in the nonrecurrence group in the training set was significantly asymmetrical—approximately 1:10; ML was applied after making the ratio of the two groups equal to 1:1 using SMOTE (Table 1). Because the volume of the data set was sufficiently large after SMOTE application, we verified the prediction model using the 20% hold-out validation method with the data partitioning of the training set and test set at 80:20 [30].

**Table 1:** Distribution of data sets before and after synthetic minority oversampling technique application.

	Training set (n=2251)			Test set (n=563)	
	Recurrence group, n (%)	Nonrecurrence group, n (%)	Recurrence group, n (%)		Nonrecurrence group, n (%)
Before	226 (10.04)	2025 (89.96)	52 (9.24)		511 (90.76)
After	2025 (50.00)	2025 (50.00)	52 (9.24)		511 (90.76)

### Statistical Analysis and ML Model Development

In this study, we compared the performance of the following representative ML classification algorithms: kernel support vector machine (SVM) [31], logistic regression [32], decision tree [33], k-nearest neighbor (KNN) [34], naïve Bayes (NB) [35], random forest [36], AdaBoost [36], and gradient boost [37]. For each algorithm, we calculated four values: sensitivity, specificity, accuracy, and area under the receiver operating characteristic curve (AUROC). The algorithm with the highest performance was finally selected based on the AUROC value, which is one of the most important indicators for confirming the performance of a classification model [38]. We used Python (version 3.7.6) for statistical analysis and algorithm development.

## Results

### Characteristics and Distribution of Patients

We compared the patient characteristics and distribution of each variable between the recurrence and nonrecurrence groups (Table 2).

The mean age of patients in the recurrence group was higher than that of patients in the nonrecurrence group (58.4 years versus 55.4 years, respectively). The average BMIs of patients in the recurrence and nonrecurrence groups were 23.6 kg/m^2^ and 24.7 kg/m^2^, respectively. The results show the same characteristics as those found in studies that have revealed better prognoses for obese patients [21]. The proportion of smokers in the recurrence and nonrecurrence groups was 25.5% and 20.1%, respectively. The pathology stage—an important variable in predicting recurrence—showed that the proportion of patients with a pathological stage ≥pT2 was approximately 60.4% (168/278) in the recurrence group and 15.2% (386/2536) in the nonrecurrence group. Approximately 77.7% (216/278) of the patients in the recurrence group and 44.8% (1135/2536) of those in the nonrecurrence group had Fuhrman nuclear grades ≥3; thus, the recurrence group had higher Fuhrman nuclear grades. The distribution of each category of pathological variables is shown in Table 2.

**Table 2:** Baseline characteristics of patients (N=2814).

Variable			Recurrence group (n=278)		Nonrecurrence group (n=2536)
Age (years), mean (SD)			58.4 (11.9)		55.4 (12.7)
BMI (kg/m^2^), mean (SD)			23.6 (3.2)		24.7 (3.3)
**Gender, n (%)**					
	Male	212 (76.3)		1811 (71.4)	
	Female	66 (23.7)		725 (28.6)	
**Smoking, n (%)**					
	Nonsmoker	207 (74.5)		2026 (79.9)	
	Current smoker	71 (25.5)		510 (20.1)	
**Pathological tumor stage, n (%)**					
	1a	50 (18.0)		1663 (65.6)	
	1b	60 (21.6)		487 (19.2)	
	2a	30 (10.8)		106 (4.2)	
	2b	12 (4.3)		29 (1.1)	
	3a	82 (29.5)		201 (7.9)	
	3b	34 (12.2)		36 (1.4)	
	3c	1 (0.4)		3 (0.1)	
	4	9 (3.2)		11 (0.4)	
**Histologic type, n (%)**					
	Clear cell	242 (87.1)		2243 (88.4)	
	Papillary	14 (5.0)		44 (1.7)	
	Chromophobe	4 (1.4)		180 (7.1)	
	Collecting duct	5 (1.8)		4 (0.2)	
	Unclassified	5 (1.8)		15 (0.6)	
	Multilocular cystic	0 (0.0)		19 (0.7)	
	Mixed	6 (2.2)		24 (0.9)	
	Xp11.2 translocation	1 (0.4)		3 (0.1)	
	Clear cell papillary	1 (0.4)		4 (0.2)	
**Necrosis, n (%)**					
	No	143 (51.4)		2272 (89.6)	
	Microscopic	30 (10.8)		126 (5.0)	
	Macroscopic	105 (37.8)		138 (5.4)	
**Lymphovascular invasion, n (%)**					
	No	200 (71.9)		2436 (96.1)	
	Yes	78 (28.1)		100 (3.9)	
**Capsular invasion, n (%)**					
	No	148 (53.2)		2114 (83.4)	
	Yes	130 (46.8)		422 (16.6)	
**Fuhrman nuclear grade, n (%)**					
	1	5 (1.8)		108 (4.3)	
	2	57 (20.5)		1293 (51.0)	
	3	141 (50.7)		1008 (39.7)	
	4	75 (27.0)		127 (5.0)	

### Prediction Model Performance

We trained eight ML algorithms on the training data set and calculated the sensitivity, specificity, accuracy, and AUROC values using the test data set (Table 3). The NB algorithm showed higher performance than the other algorithms, with an AUROC of 0.836 within 5 years and 0.784 within 10 years. The NB approach calculates the conditional probability, which is the likelihood that a conclusion will be observed based on the evidence given [35]. The NB algorithm is simple and fast [39] and has proven effective in text classification and medical diagnosis [40,41]. However, the NB approach has a limitation in that its prediction probability becomes zero when a new value that is not in the training data set is entered; Laplace smoothing is a means of solving this problem [42]. The predictive model we developed also had a problem in that the probability value became zero when a new type of data that was not in the training data set was entered; hence, the algorithm was optimized by adjusting the α value—a parameter in Laplace smoothing (Table 4).

**Table 3:** Diagnostic performance of machine learning algorithms for the prediction of renal cell carcinoma recurrence.

Algorithm (parameter name) and parameter value (in 5 years, in 10 years)		Sensitivity		Specificity		Accuracy		AUROC^a^	
		5-year	10-year	5-year	10-year	5-year	10-year	5-year	10-year
Kernel SVM^b,c^		0.733	0.673	0.805	0.853	0.800	0.837	0.769	0.763
Logistic regression^c^		0.644	0.692	0.839	0.816	0.823	0.805	0.741	0.754
Decision tree^c^		0.533	0.442	0.866	0.869	0.839	0.829	0.700	0.656
**KNN^d^ (n-neighbors)**									
	(100, 100)^c^	0.556	0.519	0.905	0.898	0.877	0.863	0.730	0.709
	(10, 10)	0.467	0.426	0.947	0.928	0.909	0.881	0.707	0.675
	(50, 50)	0.511	0.461	0.931	0.922	0.898	0.879	0.722	0.692
	(200, 200)	0.556	0.481	0.899	0.902	0.871	0.863	0.727	0.691
**NB^e^ (alpha)**									
	(10, 100)^c^	0.822	0.731	0.850	0.828	0.848	0.819	0.836	0.784
**Random forest (number of trees**)									
	(5, 5)^c^	0.578	0.500	0.858	0.853	0.835	0.821	0.718	0.677
	(10, 10)	0.511	0.423	0.866	0.861	0.837	0.821	0.688	0.642
	(50, 50)	0.511	0.442	0.875	0.861	0.846	0.822	0.693	0.652
	(100, 100)	0.511	0.462	0.864	0.861	0.835	0.824	0.687	0.661
**AdaBoost (number of trees)**									
	(50, 200)^c^	0.733	0.692	0.815	0.810	0.809	0.800	0.774	0.751
	(10, 10)	0.600	0.577	0.895	0.845	0.871	0.821	0.747	0.711
	(50, 50)	0.733	0.673	0.815	0.824	0.809	0.810	0.774	0.748
	(100, 100)	0.711	0.692	0.835	0.802	0.825	0.792	0.773	0.747
	(200, 200)	0.711	0.692	0.837	0.810	0.826	0.800	0.774	0.751
**Gradient boost (number of trees)**									
	(50, 100)^c^	0.688	0.635	0.819	0.826	0.809	0.808	0.754	0.730
	(10, 10)	0.756	0.596	0.667	0.849	0.674	0.825	0.711	0.723
	(50, 50)	0.688	0.615	0.819	0.826	0.809	0.806	0.754	0.721
	(100, 100)	0.555	0.635	0.823	0.826	0.805	0.808	0.711	0.730
	(200, 200)	0.533	0.558	0.848	0.832	0.823	0.806	0.691	0.695

^a^AUROC: area under the receiver operating characteristic curve.

^b^SVM: support vector machine.

^c^Final algorithms selected by adjusting parameters.

^d^KNN: k-nearest neighbor.

^e^NB: naïve Bayes.

**Table 4:** Performance according to the α value in the naïve Bayes model.

α value	Sensitivity		Specificity		Accuracy		AUROC^a^	
	5-year	10-year	5-year	10-year	5-year	10-year	5-year	10-year
0 (no smoothing)	0.800	0.731	0.848	0.828	0.844	0.819	0.824	0.779
1	0.822	0.731	0.848	0.828	0.846	0.819	0.835	0.779
10	0.822	0.731	0.850	0.834	0.848	0.824	0.836	0.782
20	0.800	0.731	0.850	0.834	0.846	0.824	0.825	0.782
30	0.800	0.731	0.852	0.834	0.848	0.824	0.826	0.782
100	0.800	0.731	0.854	0.840	0.850	0.828	0.827	0.784
200	0.756	0.692	0.860	0.845	0.852	0.831	0.807	0.769

^a^AUROC: area under the receiver operating characteristic curve.

**Figure 2:**
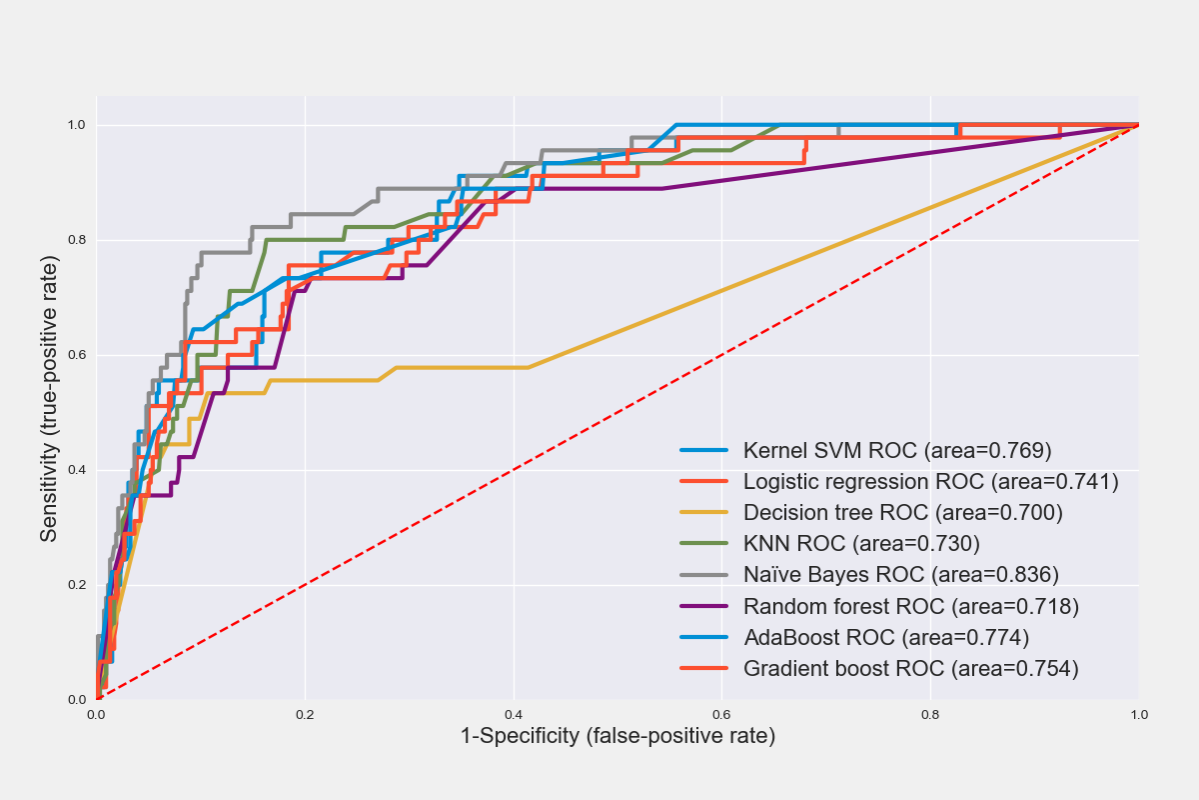
Receiver operating characteristic (ROC) curves of recurrence prediction algorithms within 5 years. KNN: k-nearest neighbor; SVM: support vector machine.

**Figure 3:**
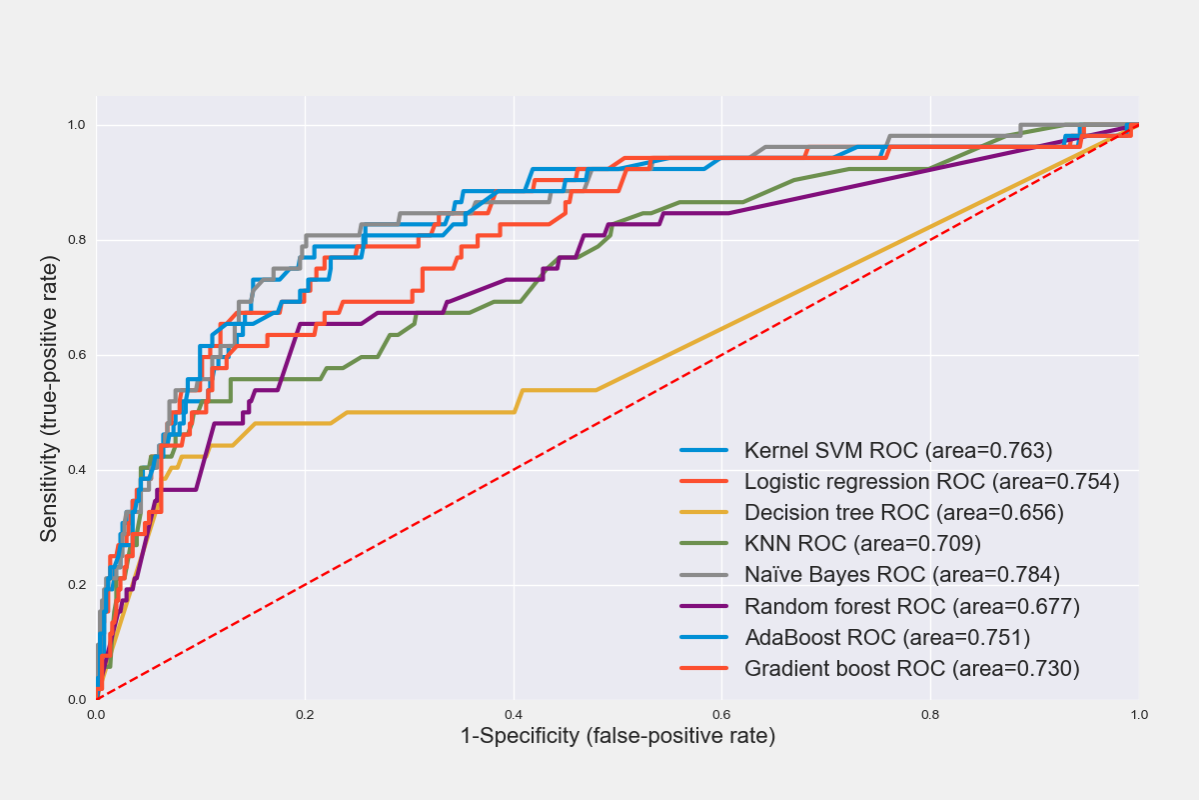
Receiver operating characteristic (ROC) curves of recurrence prediction algorithms within 10 years. KNN: k-nearest neighbor; SVM: support vector machine.

For predictions within 5 years, the AUROC was found to be 0.836 when α=10, which was the highest performance compared with that before smoothing was applied (α= 0, AUROC 0.824). For predictions within 10 years, the AUROC was 0.784 when α=100, which was the highest performance compared with that before smoothing was applied (α=0, AUROC 0.779). When comparing the area by drawing the ROC curve of the prediction algorithm within 5 and 10 years, the NB curve line was close to the upper left corner, which means that the area for that algorithm was the widest (Figures 2 and 3).

## Discussion

### Principal Findings

In this study, we developed an algorithm to predict the probability of RCC recurrence within 10 years by selecting 10 variables that significantly affect recurrence. The AUROC of the algorithm was 0.84 for models of recurrence within 5 years and 0.79 for models of recurrence within 10 years. Our proposed algorithm achieved better prediction performance than the previously developed 5-year prediction algorithm by MSKCC, which yielded AUROCs of 0.74 [14] and 0.82 [15].

In the previous studies, 66 recurrences in 601 patients [14] and 72 recurrences in 701 patients [15] were used to form the data set for analysis. Because the data were collected from a single institution, the scale was small, and the data included censored data. The methods that can be applied to analyze censored data are limited. Therefore, in previous studies, an algorithm was developed using the Cox proportional hazards model—the most representative survival analysis method—and its performance was presented.

Because the results of previous studies were based on a single institutional analysis, the characteristics of patients in various regions were likely not reflected, meaning biased results may have been obtained. Thus, a data set composed of data from eight institutions in various regions of Korea was used in this study. In our data, 278 out of 2814 patients experienced RCC recurrence, and censored data were not included. We attempted to improve the prediction performance using more diverse and significant variables than those used by the prediction algorithms in previous studies. Finally, we developed a prediction algorithm by applying ML techniques that are typically used in classification tasks. Because we used large-scale data that sufficiently reflect the characteristics of patients with RCC in Korea, the proposed algorithm achieved stable results with high accuracy and low bias.

To the best of our knowledge, this is the first study to predict the recurrence of RCC within 10 years after surgery using ML techniques. The recurrence of most cancers is typically within 5 years. Because RCC has a late recurrence [12], it is vital to predict the late recurrence in advance and establish a personalized treatment strategy for managing the prognosis of patients with RCC. Thus, our study makes an important contribution by accurately predicting the likelihood of late recurrence of RCC.

### Limitations

We utilized the data of patients with RCC recurrence after 1 to 10 years in the recurrence prediction model within 10 years. However, in several studies, a difference between variables that affect early recurrence and late recurrence was observed [12,43]. Therefore, the prediction models for 1 to 5 years and 5 to 10 years should be distinct from each other and should be constructed using different combinations of variables. However, despite being a large cohort representing the whole of Korea, it was difficult to create a single model, as only 23 cases occurred after 5 to 10 years. Therefore, in this study, we developed a predictive model by integrating both groups within 10 years. Hence, the algorithm for within 10 years seems to have lower performance than the model for within 5 years because of the heterogeneity between the 1- to 5-year recurrence group and the 5- to 10-year recurrence group. We plan to develop additional stable and accurate models to predict late recurrence when data are collected after 5 to 10 years.

Furthermore, we used large-scale cohort data showing the characteristics of patients with RCC in Korea. Therefore, the algorithm we developed exhibits stable performance when applied to Korean patients with RCC. However, patients with RCC have different demographic and clinical characteristics; hence, the performance may be reduced when applied to different ethnicities [44,45].

### Conclusions

Using the KORCC database, a large-scale cohort of RCC in Korea, we developed an algorithm to predict the probability of RCC recurrence after surgery using a representative ML technique. Among the eight ML algorithms, the NB algorithm showed the best diagnostic performance in both the 5-year model and the 10-year model in terms of the AUROC. The developed algorithm can help clinicians establish postoperative prognosis management and personalized treatment strategies for patients with RCC.

## Abbreviations

AUROC: area under the receiver operating characteristic curve
KNN: k-nearest neighbor
KORCC: KOrean Renal Cell Carcinoma
ML: machine learning
MSKCC: Memorial Sloan Kettering Cancer Center
NB: naïve Bayes
RCC: renal cell carcinoma
SMOTE: synthetic minority oversampling technique
SVM: support vector machine
